# The Child in Dentistry

**Published:** 1904-10

**Authors:** Bessie Burns Bennett

**Affiliations:** Baltimore, Md.


### THE CHILD IN DENTISTRY.

By Bessie Burns Bennett, D. D. S.,
Baltimore, Md.

(Written for *The American Journal of Dental Science.*)

What is the relation of the child to the dental profession at large?

Similar to that of the acorn to the oak, or the tiny grains of wheat to the full field of golden splendor, for do not the children of today represent the men and women of coming years, the parents of a future generation; and upon the manner of their development does not the moral standing and the physical welfare of a coming race depend?

So, if the child of today be *well* trained in the value and care of the contents of its oral cavity, blessed is the dentist who may have the care of that child through its childhood and maturity; but if the child be badly trained, or not at all in these matters, woe to both patient and practitioner in the days to come.

A good motto for the dentist's sanctum is

"Honor and fame from no condition rise,
Act well *your* part, therein the honor lies."

*"Act well your part."* If every dentist were to carry this out in the spirit as well as the letter, instructing each patient who is a parent, pointing out what is best for themselves as also for the little beings entrusted to their love and care, emphasizing what might seem trivial matters—for too often the trivial matters form the keynotes in the grand chorus of success; and Michael Angelo knew whereof he spoke when he said, "Perfection is made up of trifles and perfection is no trifle." If the parents *act well their part* toward the little ones, guiding the tiny hand till it becomes expert in the use of the tooth brush, carefully scanning the teeth for the slightest evidence of decay, zealously guarding the child from outrageous tales of ill-treatment and cause of fear at the hands of the dentist.

If the dentist himself act toward the child the part of a gentleman in the old English sense of the word, in all respects doing to the little one just as he would have done to himself under like circumstances, think you there would be septic mouths presented so often and a race seventy-five per cent of whom dread the dental chair to such a degree that the mere anticipation of dental operations causes suffering?

One little man under my care is a highly nervous, delicate little mortal, whose teeth show the ravages of continued ill-health and constant dosing, and above all things he hates the dentist's chair—or did when he came to me.

By judicious treatment in always doing just what I promise, by explaining why certain operations must necessarily be painful, by short sittings, and above all by keeping up a continued strain of conversation, spiced with funny stories or anecdotes, I have won over the little fellow so much from his antipathy, that although he is still at a far point from *loving* dental operations he comes to me without that dread which so racks little nervous systems, indeed sometimes adults suffer serious illness in the anticipation of the "torture-cabinet," as I heard one once term a dental office.

Another little patient is a tiny maid of five, whose first introduction to the dental chair was through me.

Now with children (and I know some of our older brethren will disapprove) I always begin right away with the engine, because if they see that mamma has it used on her teeth, and it is tabooed for them, their quick wits reason it to be something dreadful, so when it is *necessary* to use the engine there's trouble right there for both parties.

So as usual I made preparations for the engine, telling my small patient that if I hurt her she could raise her hand, and I would immediately stop.

The bur wa still about twelve inches distant from the youngster's mouth, but up went the arm full length, carrying out the maxim probably, "In time of peace prepare for war."

However in a few seconds I showed the little one that unless she jumped, "that thing," as she dubbed the bur, could only hurt in one place at a time, and that was all she had to think about as I had promised to stop if she said so.

The little lady saw the practicability of this reasoning, and although I knew some of the cavities were somewhat sensitive, she gave me no trouble whatever. In fact she is so pleased with her dentist that she has dropped all conventionality in addressing me and insists on coming with every member of the family who has an appointment. She recently asked her mother if she didn't think the baby (a little over two) had better be taken to me to have her teeth cleaned, as "they are looking dark."

Children are taught that it is the height of ill-breeding to be seen with soiled hands, faces, nails or unbrushed hair, yet many children can be found carrying in their mouths (through which all nutriment for the body must pass and become contaminated) enough dirt, and very dirty dirt, charged with pathogenic as well as harmless bacilli; enough dirt, I say, which if displayed on hands or faces would fill even the most careless parent with dismay.

And yet it is social law which demands clean faces and hands, so the children must needs adapt themselves to circumstances and appear clean, but it is *health* demands that the children's mouths be clean and yet—?

If the child be properly trained in the keeping of the oral cavity and its contents sweet and clean such habits will become second nature, and he will no more appear in public with ill-smelling breath and teeth covered with sordes than with his face decorated with a corresponding amount of virgin soil.

But as long as mothers neglect and dentists condone the fact that the little ones present themselves at intervals with accumulations of tartar and debris, just "slow up" and let the miscreants go on their happy way, instead of reading the law to them added to some salient facts which the small patients would do well to digest, or better still a sharp home thrust to the youngster's pride. So long as the dentist allows the misdemeanor of an unclean mouth to recur again and again, just so long will the child continue to become closer and closer involved in the toils of the bad habit, until when remonstrance *does* arrive it is almost in vain, and as long as this state of affairs continues, so likewise will the dental mechanism of the race continue to deteriorate, just as the magnificent dentures of the North American Indian have been influenced by contact with civilized customs.

Aside from the lowering of the moral standard, let us see what ill-effects an unclean mouth and carious teeth may produce.

To start with, caries alone causes endless pain in a child's mouth, the pulp being so large in proportion to the tooth: the beauty of the little face is marred by blackened, decayed stumps revealed by speech or laughter; the breath which should be sweet as the fragrant dews is charged with evil-smelling gases; the mechanism of the stomach is sadly overwrought by having to digest comparatively unmasticated food thrown into it because the child prefers to bolt its food rather than suffer pain in the process of mastication. In this way serious gastric or intestinal trouble may be brought about.

Then again, through the medium of such an admirable culture field as an unhygienic oral cavity, the child may be inoculated with diseases peculiar to childhood, which, under different circumstances, it might have escaped.

Then, too, the child not being trained properly in oral hygiene, will continue in its neglect until maturity is reached, when probably the penalty will be paid in some dread mental disorder, resulting from accumulations of tartar, lack of use and general sepsis, or at best the beautiful handiworks of Nature will be superseded by bridge work or "a plate."

A living illustration of this is now under my treatment, who says he has never cleaned his teeth more than ONCE A WEEK!!!

Hercules' task of cleaning the stables of Augeus could not have much surpassed the cleaning of that patient's teeth.

But, "Patience," it has been said, "is the strength cheerfully to wait and courageously to work regardless of delay and disappointments."

If this be the true definition, patience is the quality desired in this connection,—hand in hand, however, with her sturdier sister "Perseverance, — "that character which impels one steadfastly to pursue the object in view with an invincible determination to triumph over all opposition, "for with *'patience' alone* we might continue 'cheerfully' to wait and courageously to work," and the "patients" would triumph in all probability over us.

It is a small matter to wield the toothbrush three times a day; still less trouble to rinse the mouth thoroughly with some slightly stimulating wash. And how many times is one repaid by the sweet, clean feeling of mouth and teeth afterward!

If in all this controversy as to whether the three years' course is best for the student, or whether the four years' course is best,—or whether the National Association of Dental Faculties had the prerogative to make a change back to the three years without the years notice called for in its constitution, if in all this agitation, I say, there had been some provision made somewhere in the course, let it be then a four years, for none of the *practical* instruction for the student in the management of young patients, and the training of them in habits of oral hygiene, some good might have accrued, for as matters now stand, I am afraid very little PRACTICAL instruction is given on this vital point, and theory, when applied to practice, often acquires characteristics which are not bargained for.

And why shouldn't there be a *chair* in dental colleges on the treatment of children?

Dentistry has progressed too rapidly to still hold that the methods applied to the adult are suitable for the child. But, as I have said before, all this can not be accomplished in a day, a week, or month, but Patience must sit enthroned for many moons before we or our successors can see the results of our efforts, and we must remember—

"The heights by great men reached and kept
Were not attained by sudden flight,
But they, while their companions slept,
Were toiling upward in the night."

It pleases me to note that our confréres in Sweden are toiling upward through the clouds which encompass this lack of hygiene, and they contemplate a popular distribution of some simple laws for the care and value of the teeth.

America is conceded to be the most progressive nation of the world. Why can't we, as dentists, fall in line with this movement and have formulated some simple rules for our clientele which the youngest patient could understand, telling of the inestimable value of the teeth, how and how often to clean them, how to watch the teeth so as to detect the first symptoms of decay, how to detect the deposition of tartar on buccal and lingual surfaces, how to use the floss or, if a toothpick *must* be used, how to prepare a *quill* one, explaining why *wood* or a *pin* should never be used? In short, embodying all the requirements for the perfect asepsis (as far as is possible) of the human mouth, couching them in simple yet forcible terms,—impressing upon the recipient that the rules are for *action*, not *contemplation*, a-la Kipling,—

Now these are the laws of the dentist,
And simple and few are they.
And the head of the law is the toothbrush,
And the strength of the law is "O-B-E-Y."

---

Tyree's Antiseptic Compound is a powder which combines strong antiseptic powers with an exceptionally agreeable odor.

It is used in the proportion of a large teaspoonful to a pint of water for cleansing discharging mucuous membranes and the dentist will find it a valuable help in cases of this class.

### INCIDENTS OF PRACTICE.

By W. A. L. Knowles, M. D., D. D. S., San Francisco.

(Written for *The American Journal of Dental Science.*)

